# A Treatise on the Circulation of the Blood

**Published:** 1834-10-01

**Authors:** 


### A TREATISE ON THE CIRCULATION OF THE BLOOD. By *J. F. Handley*. Chipping Norton. Octavo, pp. 33. 1834.

WE have neither much space nor patience to devote to a review of this strange production, although every page might afford room for ill-natured criticism to the philologist, as well as to the physiologist. Here is a sample of the whole—a brick, which may be taken as a specimen of the building. At page 14, the reader is informed—

"I do not hesitate, therefore, to say, that atmospheric air is necessary to be received into the lungs, not to stimulate the fibres of the heart and bloodvessels, but to re-establish the currency of the fluid, after it has become effete by passing through the different channels of the body; that the force with which it circulates is regained at the commencement of the pulmonary veins, and completely exhausted at the termination of the pulmonary arteries; and that the effect derived from the mixture of atmospheric air with the blood is not to give it stimulum, but to blend with it an elastic and expansive force, by which means it obtains its moving impulse; but in what manner I shall attempt hereafter to explain." 15.

It would seem, from the tenor of this sentence, as well as from some remarks further on, that the blood, during its pulmonary circulation, becomes completely saturated, to the maximum point, with air, which is absorbed, in our author's opinion, in immense quantities, and imparts a high elastic resistance to the arterial mass; and that, in short, it is in the state of a powerfully-condensed or compressed vapour or gas, ready to dilate itself whenever the pressure is removed. At page 22, the announcement is made—

"That every particle of blood in the whole body, by repeated exposures, becomes impregnated with atmospheric air, inasmuch as it maintains an affinity for it; and that according to the diminution of the number of the vessels which contain it, so will be the increase of its velocity, that is to say, its force will be concentrated by accumulation; for if the elastic force of the fluid contained in each vessel of the rete mirabile, supposing that there are 1000 vessels, be as one, then the same force as it flows through the pulmonary veins, being reduced to four in number, will be 250 times greater than in each vessel of the rete mirabile; and when it arrives at the left auricle of the heart, being there contained in one vessel, its force will be a thousand times greater: and hence it will appear how absolutely necessary it is that the left auricle and ventricle of the heart (but particularly the ventricle, for that alone receives the force of its entire accumulation) should be well fortified against such an impetuous rush of the fluid into their cavities. It is on this account that the strong muscular fibres of the heart are given, that is to resist, and by their resilience, throw back the force of the fluid upon itself, so as to propel it forward till it gradually loses its force in distribution; and thus according to this theory, the greatest velocity of the blood will be in the left auricle and ventricle of the heart—the least in the termination of the arteries." 22.

Then follows the explanation of the circulation in the veins. The key to the explanation is in the following sentence.

"Every solid substance or liquid portion of matter will gravitate to a fixed point is a well-known fact; therefore we will suppose the whole body to be perpetually gravitating towards its centre, that is, every superficial part of it is pressing in that direction ; now allowing this to be the case, as indeed it is, the lighter parts will give place to the heavier, so that the blood which flows through the vessels of the skin will actually receive its weight. Now as the skin and different organs of the body are specifically heavier than the blood itself, it must necessarily follow, that the blood will be pressed by them in a direction contrary to that in which the force of such pressure is applied to it, so that it must either flow forwards into the veins, or backwards into the arteries again." 23.

This is a most unlooked-for application of the doctrine of centripetal forces to the science of physiology ; and the precision of anatomical facts is made subservient, by our author's plastic mind, to the confirmation of his peculiar views. As the thickness and strength of the left ventricle and auricle of the heart are supposed to be indicative of the very powerful expansive or elastic force of the aërated blood, so it is ingeniously imagined by our fanciful author, that the right side cavities are of considerable thickness and strength, for the purpose of being fortified against the force of the gravitating venous blood. But enough of these reveries.

Some of the words and phrases of Mr. Handley deserve a new dictionary on their own account—for example, " sarcous substance," " currency of the blood," " the fibres retort the fluid," " refellent," " energizes," &c. Alas! for the cacoethes scribendi !

